# Supplementary material for: “We usually see a lot of delay in terms of coming for or seeking care”: an expert consultation on COVID testing and care pathways in seven low- and middle-income countries
Source: BMC Health Serv Res. 2023 Nov 23;23:1288. doi: 10.1186/s12913-023-10305-0 (PMC10666325; doi:10.1186/s12913-023-10305-0)
Supplement: Supplementary file 1 — Additional file 1. [file 12913_2023_10305_MOESM1_ESM.zip › Interview guide.docx]

Experts’ Meeting on COVID-19 Care Pathways Supported by Rapid Diagnostic Tests (RDTs)

Interview guide

This document outlines the structure/guiding questions for the semi-structured interviews undertaken with regional groups of experts.

# Background

*Presentation of the objectives of the meeting and general reminders.*

The long-term objective of these meetings is to inform an assessment of the cost-effectiveness of RDTs to support COVID treatment and care in LMICs. This work is informed by:

- Data collection (e.g., of the cost of COVID-19 testing in different contexts).
- Literature review (to get estimates of parameter ranges).
- These experts’ meeting, which aim at:
  - Promoting a better understanding of care pathways.
  - Getting some sense of ranges for badly informed parameters.

There will be two regional meetings with experts from your region and one summary joint meeting where you will be able to hear about the results from across all regions.

As explained in the information sheet and consent form, data will only used in an aggregate manner (individual responses will be anonymized and reported so that they cannot be linked to you).

We will address the following themes:

At the first meeting:

- Population subgroups to consider in analysis.
- Screening and testing practices with/without RDTs.
- Testing priorities.
- Linkage to care for self-testers.

At the second meeting:

- Monitoring, treatment and care practices at health centers with/without RDTs.
- Expected changes in 2023.
- Any other issue related to care pathways or data.

# Introductions

*Participants introduce themselves briefly.*

# Exploratory questions

*These questions may be used to prompt further conversation on each of the subjects we want to address. The discussion will also be informed by the written documentation sent in advance by participants.*

**First meeting**

1. *Population groups of interest*

Could you please list the population subgroups of particular interest to policymakers in the context of COVID e.g., people living with certain comorbidities, hard-to-reach groups, etc. that would benefit from being modelled separately? For example:

- Are some comorbidities or profiles particularly represented among at-risk individuals with a COVID infection in your country?
- Are there subgroups likely to be particularly under-/over-represented in visits to care centers and/or among self-testers?
- Is their risk status likely to be accurately identified?
- What data/information sources are available in this regard, if any?

1. *Screening*

Thank you for sending us your countries’ COVID screening guidelines. We would like to better understand how patient screening for COVID and/or to identify individuals at higher risk from COVID takes place in practice:

- How is screening conducted in practice? Which patients are screened, at what point in the care pathway and where, by whom?
- Are the required resources/tools available to screen for symptoms (e.g., oximeters)?
- If not, what are the consequences e.g., under-identification/treatment of severe cases? Do you have any data on this?

1. *Testing*

Thank you for sending us your countries’ testing guidelines and protocols. We want to better understand what the testing practices for screen-positive patients are and what modalities of rapid diagnostic testing are most important for us to assess in a cost-effectiveness analysis.

- What RDT uses would be most important to reflect in cost-effectiveness analyses to inform policymaking e.g., provider-led, self-administered, in what contexts? If self-testing is of high interest, what forms is it expected that it would take e.g., testing of contacts, availability in drug shops, etc.?
- Any data on practices regarding confirmatory or repeat testing (any challenge to implementing guidelines in practice)?
- What is the standard of care in the absence of RDTs (e.g., clinical assessment; PCR onsite/offsite, what is the timeframe for PCR results to be obtained)? Does it differ by patient group?
- What groups would be prioritized (e.g., severe, high-risk) if RDTs had limited availability?

1. *Self-testing and linkage to care*

What do we expect self-testers to do once they get a positive or negative test?

- - What is the expected pathway of care for self-testers that test positive? How often do we expect self-testers to seek care? What therapeutics are they likely to take for their symptoms at home? Are there data sources for this?
  - What is the expected pathway of care for self-testers that test negative? How often do we expect self-testers to seek care for their symptoms? Repeat testing? What therapeutics are they likely to take for their symptoms at home? Are there data sources for this?
- If someone has no access to self-testing at home, what self-medication are they likely to take? At what point are they likely to seek care? Is there any data on this?

**Second meeting**

1. *Treatment and care at health centers*

Thank you for sending your countries’ treatment and care guidelines. We want to better understand what care pathways are used in practice for confirmed positives, confirmed negatives, or suspect cases that cannot be tested:

- What range of monitoring, treatment and care options are commonly available for confirmed cases at different levels of severity/risk? What options are unlikely to ever become available in the future?
- Are any COVID therapeutics not recommended or recommended against by WHO (see [WHO recommendations on their website](https://www.who.int/publications/i/item/WHO-2019-nCoV-therapeutics-2022.5)) likely to be given to positive patients? How commonly? Is there any data source about that?
- What happens if a suspect case cannot be tested? What treatment or care would they be provided for different severity/risk levels?
- What is the testing/care pathway for people with a negative diagnosis?
- Are there circumstances in which you would still treat for COVID?
- What is the most common disease that could be confused with COVID in your context?

1. *Other issues and evolutions in 2023:*

- In light of our discussion, which of the parameter estimates we provided in appendix appear realistic and are there any that you would like to see adjusted? Why? We would like to particularly focus on:
  - The sensitivity and specificity of clinical screening
  - The impact of a positive or negative test result (or of no result) on antibiotic use.
- Are important changes expected in 2023 in terms of e.g., the availability and cost of different tests, screening tools and therapeutics? Do we have sources for that information?
- Are there any other issues we have not yet discussed that needs to be addressed?

# Closing and next steps

Thank you very much for your great contributions. Following the regional meetings you attended, we will also have, as planned, a joint meeting. This will be an opportunity for you to hear about other regions. This work will also feed into papers to which you will have an opportunity to be co-authors.
