# Supplementary material for: “We usually see a lot of delay in terms of coming for or seeking care”: an expert consultation on COVID testing and care pathways in seven low- and middle-income countries
Source: BMC Health Serv Res. 2023 Nov 23;23:1288. doi: 10.1186/s12913-023-10305-0 (PMC10666325; doi:10.1186/s12913-023-10305-0)
Supplement: Supplementary file 1 — Additional file 1. [file 12913_2023_10305_MOESM1_ESM.zip › Protocol experts' meeting RDT care pathways - Final.docx]

Experts’ Meeting on Rapid Diagnostic Test-Supported COVID Care Pathways – Protocol

**Organisers:** Gabrielle Bonnet^1^, Liz Corbett^3^, Mark Jit^1^, Anna Vassall^2^

^1^ Department of Infectious Disease Epidemiology, Faculty of Epidemiology and Population Health, London School of Hygiene & Tropical Medicine

^2^ Department of Global Health and Development, Faculty of Public Health and Policy, London School of Hygiene & Tropical Medicine

^3^ Department of Clinical Research, Faculty of Infectious and Tropical Diseases, London School of Hygiene & Tropical Medicine

# **Summary**

COVID policies are evolving and increasingly aiming at reducing disease burden. Rapid diagnostic tests (RDTs) can play an important part in these policies because of their ability to expand testing and provide faster linkage to care. In this context, it is important to understand the cost-effectiveness of RDT-supported COVID care pathways. This requires the design of COVID screening, testing and care pathways that reflect local constraints. This expert’s meeting will seek low-and-middle-income country experts’ insights on population groups and screening, testing, treatment and care practices. There will be a number of regional meetings where experts will discuss most common practices and ranges and/or suggest information sources, followed by a joint meeting with all regions. The meetings will be focus group discussions informed by documentation (e.g., national guidelines/protocols on COVID screening, testing and care, self-testing uptake/linkage to care information sources) collected in advance. We will follow research ethics procedures as the intention will be to publish the findings of the meetings as well as use these findings in further analyses. All participants will be asked written consent and consultation results will be published as anonymized summaries to prevent identification of individual contributions.

# **Background**

As COVID has shifted to a high-burden endemic phase, countries’ policies have evolved from transmission reduction to burden reduction strategies. Rapid diagnostic tests (RDTs) may help with that purpose, both through self-testing and/or expansion of professional-administered testing, but their role often remains unclear. The ongoing development of promising therapeutic options (such as antivirals), some of which require early diagnosis and treatment, is driving interrogations regarding the ways in which RDTs may best support COVID care efforts in low-and-middle-income countries (LMICs).

In this context, we are organizing an experts’ consultation regarding LMIC practices, data and priorities. This will support the future development of a cost-effectiveness analysis of RDT-supported COVID care pathways in LMICs. This consultation will seek to understand which patient groups are of particular interest and which screening, testing and care pathways are realistic for patients with COVID-like symptoms both with and in the absence of RDTs. As the availability and cost of care options may evolve rapidly, we also need to understand if important changes are foreseen in the coming year.

# **Justification**

Population subgroups are an important issue: while they are in part determined by global data on the symptoms, comorbidities, ages or immunity status associated with severe outcomes and by the benefits of existing therapeutics for different patient profiles [1-3], which risk profiles are most common among infected individuals and those seeking care depends on the country/region. In turn, the comorbidities and ages of tested high-risk individuals affect the years of life gained through treatment.

We also need to understand screening and testing practices. Both the screening tools used [4-8] and the modalities of their use may vary. Further, some of the tools that can support screening (such as oximeters) may be lacking [9-12]. There can therefore be a broad range in screening sensitivity and specificity rates. There may also be some variability in testing practices for screen positives, particularly for people who self-test (e.g., repeat/confirmatory testing) or in contexts of constrained test availability.

Finally, once a patient is diagnosed, global guidance e.g., the WHO list of therapeutics for COVID-positive cases [3] provides information on recommended therapeutics depending on symptom severity and risk level. Candidate treatments that are recommended against, or which efficiency has not been proven yet, are also listed. In practice, availability of COVID treatments varies by country and association of care such as oxygen therapy with the appropriate monitoring tools (e.g., oximeters) may also be lacking. We therefore need to understand what care pathways are realistic in the field. Care pathways include outcomes for people that screen or test negative, who may receive targeted or presumptive treatment depending on disease aetiology, availability of testing for other diagnoses and common differential diagnoses e.g., respiratory infections [14, 15] or other diseases that sometimes have a COVID-like presentation such as malaria [16].

To gain understanding of all these areas, we intend to use three data and information sources: 1) literature reviews, which will uncover what information is available or what proxies may be used, 2) field studies supported by the Africa, Asia, Americas COVID-19 Preparedness (3ACP) consortium in Malawi, Zambia and Nigeria, which will provide information on providers’ and patients’ profiles and behaviours, and 3) experts’ knowledge regarding priorities, constraints, behaviours and practices, collated through this consultation. Experts’ knowledge is particularly key to ensure the structure of the COVID care pathway used in cost-effectiveness analyses reflects practical realities in the field.

# **Objectives**

This protocol describes the purpose, tools and methodology used to seek experts’ feedback. The overall objective of the expert consultation is to support the development of an analysis of the cost-effectiveness of RDT-supported COVID care pathways in LMICs. For that purpose, we will seek to:

1. Understand what population subgroups (e.g., specific comorbidities) may need to be modelled separately; what screening/testing and care pathways are realistic and useful in different country contexts, with and without rapid testing; and whether important changes are expected in the near future.

2. Get qualitative feedback on the data obtained through literature review, and in particular if complementary or contradictory data and information sources are available.

# **Methodology**

## **Identification of experts**

We will use purposive sampling of LMIC experts (targeted number: 8-12 experts). The experts should be able to participate in a meeting in English and have a good knowledge of COVID care pathways in their countries, preferably at the community level (as opposed to major cities and health centres). Participant recruitment will be obtained by asking LSHTM contributors to projects in targeted regions and WHO partners to identify either suitable candidates or people who can identify such candidates. We wish to ensure that countries represented through these experts reflect a range of regions (Africa, Asia and America) and income levels (low-, lower-middle- and upper-middle-income countries), as well as, if possible, countries that faced different pandemic severity levels. For that purpose, if the number of potential participants exceeds the target number of participants per region (3 in Latin America, 3 in Asia and 4 in Africa), we will first contact those that would allow the greater diversity of countries in terms of income level and geographical spread. If we receive a refusal, we will ask the person to nominate another suitable expert from the same country. If not possible, we will contact other potential participants in the same region.

## **Identification of questions**

To identify the questions submitted to the experts, we have developed a draft COVID care pathway (***Figure 1***) with and without provider-led- or self-testing through RDTs, based on WHO’s list of recommended therapeutics [3]. Key uncertainties regarding the structure of the pathway (e.g., care practices in resource-constrained environments) were reflected in questions to be submitted to the experts. We are also identifying estimates for key parameters related to different stages in this draft pathway and relevant to the cost-effectiveness analysis.

## **Modalities of the consultation**

We will first send consent forms and background documentation, including the draft COVID-19 care pathway and parameter estimates based on literature review, in advance of the meeting. We will then ask participants to send key documentation relevant to the questions (any national screening, testing or treatment guidelines, and data or information on self-testing uptake and rates of linkage to care if available). This will give us a basis for the discussion and will allow us to spend more time addressing practical implementation issues. Experts will further be informed that if they have any data relating to the parameter estimates we have developed and that they want to share, they are welcome to do so.

The discussions with the experts will take place during two regional meetings of one and a half hours each. These meetings will be followed by a half hour joint meeting during which we will summarize the results from the regional meetings and major modelling implications.

The discussion during the regional meetings will be prompted by a presentation of the objectives of the meeting and of the draft COVID-19 care pathway. It will centre around the following questions:

Meeting 1:

- Population subgroups: Could you please list the population subgroups of particular interest to policymakers in the context of COVID that would benefit from being modelled separately?
- Screening: What process is used to screen patients for COVID?
- Testing: What are the rules and practices for testing screen-positive patients? In which contexts would repeat or confirmatory testing be undertaken?
- Linkage to care for self-testers: What do we expect self-testers to do after getting their test results?

Meeting 2:

- Treatment and care: What range of monitoring, treatment and care options are commonly available at health centers for patients that are confirmed positives or negatives at different levels of severity/risk? What is the standard of care in the absence of testing?
- Future changes: Are important changes expected in 2023 in terms of e.g., the availability and cost of different tests, screening tools and therapeutics?
- Other issues:
  - If we get back to the draft care pathway, are there any other issues we have not yet discussed that needs to be addressed?
  - In light of our discussion, do you think the parameter estimates we provided are realistic?

Examples of exploratory questions for focus group leads to guide the discussions are provided in **Annex 2**.

## **Funding**

This research is funded via Unitaid/PSI’s 3ACP 100584IR grant.

## **Timeline**

We expect to hold the meetings between end November and mid-December.

## **Data management, analysis and dissemination**

The outcomes of the consultation will include the recording of the sessions, a written record of the answers provided by participants (both available only to study researchers, with the recording being kept for a maximum of a month to ensure enough time to accurately transcribe the outcomes of the meetings and the written record being kept for two years) and a publication of the results of the discussion which will use only anonymized information. Co-authorship in the form of a group author will be offered to those participants that are interested. The results of the consultation will further contribute to a cost-effectiveness analysis of RDT-supported COVID care pathways in low- and middle-income countries.

## **Ethics**

Ethical approval will be sought from the relevant LSHTM Research Ethics Committee. Written consent will be sought from all participants using the information sheet and consent form in **Annex 3** prior to their participation in the study.

Benefits and harms to participants are expected to be minimal. Potential benefits include a better understanding of practices in other countries and regions and a co-authorship in the publication resulting from this study. Potential harms would arise if data were not anonymized i.e., participants might be perceived as not presenting their country or the health centres they have worked with in positively (when flagging constraints they have faced or witnessed) or the places they have worked in might be cast in a negative light. To address this risk, results will be anonymized so that no individual, institution or country can be identified.

None of the researchers or collaborators has any conflict of interest.

**Figure 1: Draft COVID care pathways**


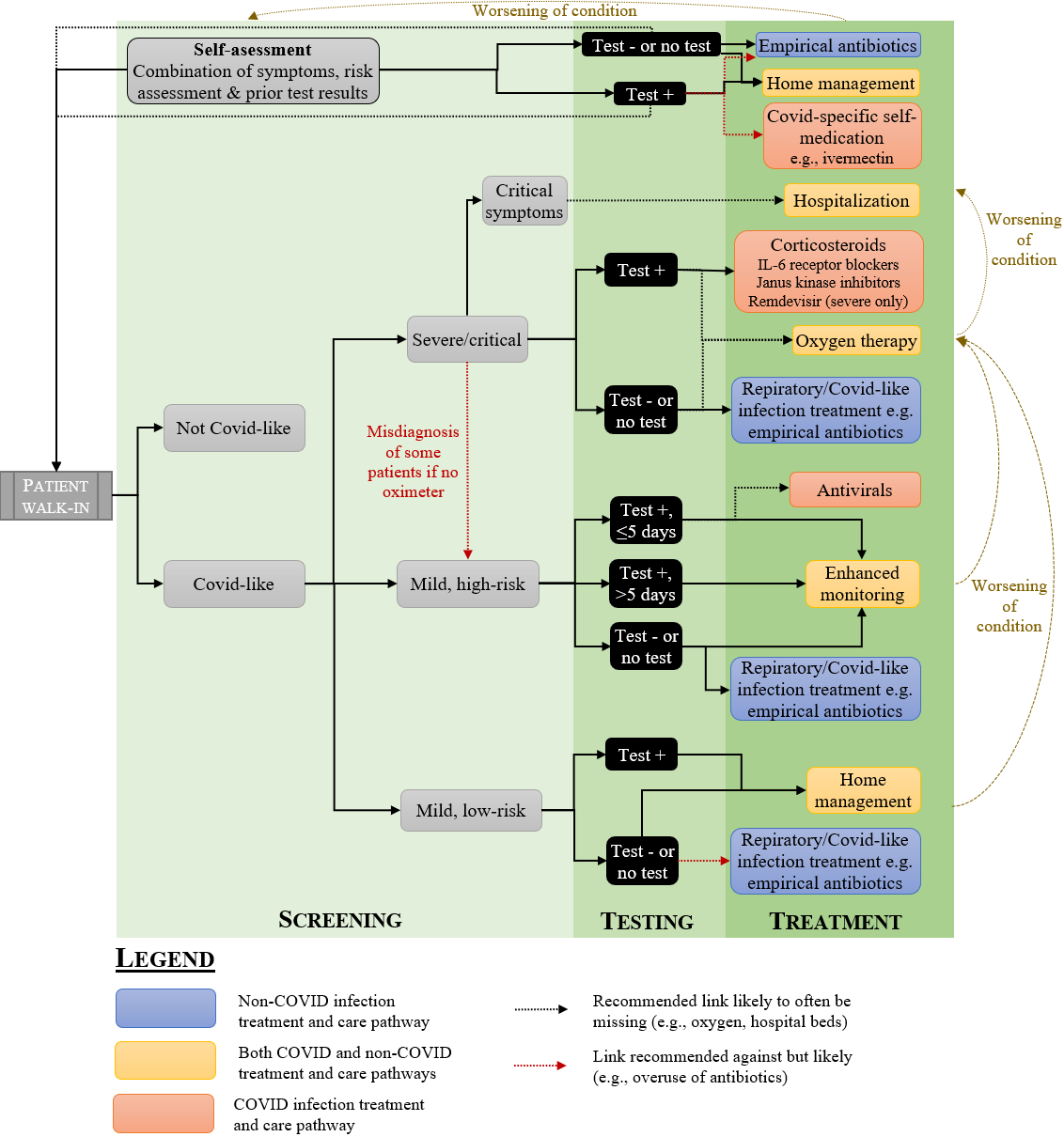


# **References**

1. Thakur, B., et al., *A systematic review and meta-analysis of geographic differences in comorbidities and associated severity and mortality among individuals with COVID-19.* Scientific Reports, 2021. **11**(1): p. 8562.

2. Zheng, Z., et al., *Risk factors of critical & mortal COVID-19 cases: A systematic literature review and meta-analysis.* The Journal of infection, 2020. **81**(2): p. e16-e25.

3. *Therapeutics and COVID-19: Living guideline, 16 September 2022.* World Health Organization: Geneva.

4. Fleitas, P.E., et al., *Clinical diagnosis of COVID-19. A multivariate logistic regression analysis of symptoms of COVID-19 at presentation.* Germs (Bucureşti), 2021. **11**(2): p. 221-237.

5. Ly, T.D.A., et al., *Screening of SARS-CoV-2 among homeless people, asylum-seekers and other people living in precarious conditions in Marseille, France, March–April 2020.* International journal of infectious diseases, 2021. **105**: p. 1-6.

6. Callahan, A., et al. *Profiling presenting symptoms of patients screened for SARS-CoV-2.* 2020; Available from: <https://medium.com/@shahlab/an-ehr-derived-summary-of-the-presenting-symptoms-of-patients-screened-for-sars-cov-2-910ceb1b22b9>.

7. Nguyen, H., et al., *376. Sensitivity and Specificity of the WHO Probable SARS-CoV-2 Case Definition Among Symptomatic Healthcare Personnel.* Open forum infectious diseases, 2021. **8**(Supplement_1): p. S290-S290.

8. Bijur, P.E., et al., *Should COVID-19 symptoms be used to cohort patients in the emergency department? A retrospective analysis.* The American journal of emergency medicine, 2022. **54**: p. 274-278.

9. PATH, *Biomedical Equipment for COVID-19 Case Management Senegal Facility Survey Report*. 2021, PATH: Seattle.

10. PATH, *Assessment Report on the Availability of Oxygen and Biomedical Equipment in Health Facilities: DRC Facility Survey Report.* . 2022, PATH: Seattle.

11. PATH, *Biomedical Equipment for COVID-19 Case Management Malawi Facility Survey Report*. 2021, PATH: Seattle.

12. PATH, *Biomedical Equipment for COVID-19 Case Management Zambia COVID-19 Treatment Facility Survey Report.* 2021, PATH: Seattle.

13. Yadav, H., et al., *Availability of essential diagnostics in ten low-income and middle-income countries: results from national health facility surveys.* The Lancet Global Health, 2021. **9**(11): p. e1553-e1560.

14. Beeching, N.J., T.E. Fletcher, and R. Fowler. *Coronavirus disease 2019 (COVID-19) (Differentials)*. BMJ Best Practice 2022 5 Sept. 2022]; Available from: <https://bestpractice.bmj.com/topics/en-gb/3000201/differentials>.

15. Hedberg, P., et al., *Clinical phenotypes and outcomes of SARS-CoV-2, influenza, RSV and seven other respiratory viruses: a retrospective study using complete hospital data.* Thorax, 2021. **77**(2): p. 1-10.

16. Heuschen, A.-K., et al., *Public health-relevant consequences of the COVID-19 pandemic on malaria in sub-Saharan Africa: a scoping review.* Malaria Journal, 2021. **20**(1): p. 339.

17. McMillan, S.S., M. King, and M.P. Tully, *How to use the nominal group and Delphi techniques.* International Journal of Clinical Pharmacy, 2016. **38**(3): p. 655-662.

18. *Group Techniques for Program Planning: A Guide to Nominal Group and Delphi Processes.* The Journal of applied behavioral science, 1976. **12**(4): p. 581-581.

19. Mason, S., et al., *Undertaking Research Using Online Nominal Group Technique: Lessons from an International Study (RESPACC).* Journal of palliative medicine, 2021. **24**(12): p. 1867-1871.

# **Annex 1: Information to be collected prior to the meetings**

We will ask participants to send us national guidelines or protocols, if there are any, associated with COVID screening, testing, monitoring, treatment and care, more specifically guidelines/protocols:

- Used to screen people for COVID and define suspect/at risk cases.
- Relative to testing, including regarding when confirmatory or repeat testing would be considered (positive/negative self-tests, severe/high risk cases, etc.).
- Relative to the monitoring, treatment and care pathway for confirmed positives, confirmed negatives, or suspect cases that cannot be tested.

We will also ask for any data that participants may have that support the discussion and future cost-effectiveness modelling i.e.:

- We will ask if records are kept or data available regarding uptake of self-testing and/or linkage to care when people are offered self-tests, or if data from other forms of self-testing that may give us insights on this are available.
- We will ask for any other data and information source that participants feel is relevant to the issues of COVID screening, testing and care in their country and that they may be able to share.

# **Annex 2 – Exploratory questions**

These questions may be used to prompt further conversation on each of the subjects we want to address during the regional meetings. The discussion will also be informed by the written documentation sent in advance by each participant.

1. *Population groups of interest*

Could you please list the population subgroups of particular interest to policymakers in the context of COVID e.g., people living with certain comorbidities, hard-to-reach groups, etc. that would benefit from being modelled separately? For example:

- Are some comorbidities or profiles particularly represented among at-risk individuals with a COVID infection in your country?
- Are there subgroups likely to be particularly under-/over-represented in visits to care centers and/or among self-testers?
- Is their risk status likely to be accurately identified?
- What data/information sources are available in this regard, if any?

1. *Screening*

Thank you for sending us your countries’ COVID screening guidelines. We would like to better understand how patient screening for COVID and/or to identify individuals at higher risk from COVID takes place in practice:

- How is screening conducted in practice? Which patients are screened, at what point in the care pathway and where, by whom?
- Are the required resources/tools available to screen for symptoms (e.g., oximeters)?
- If not, what are the consequences e.g., under-identification/treatment of severe cases? Do you have any data on this?

1. *Testing*

Thank you for sending us your countries’ testing guidelines and protocols. We want to better understand what the testing practices for screen-positive patients are and what modalities of rapid diagnostic testing are most important for us to assess in a cost-effectiveness analysis.

- What RDT uses would be most important to reflect in cost-effectiveness analyses to inform policymaking e.g., provider-led, self-administered, in what contexts? If self-testing is of high interest, what forms is it expected that it would take e.g., testing of contacts, availability in drug shops, etc.?
- Any data on practices regarding confirmatory or repeat testing (any challenge to implementing guidelines in practice)?
- What is the standard of care in the absence of RDTs (e.g., clinical assessment; PCR onsite/offsite, what is the timeframe for PCR results to be obtained)? Does it differ by patient group?
- What groups would be prioritized (e.g., severe, high-risk) if RDTs had limited availability?

1. *Self-testing and linkage to care*

What do we expect self-testers to do once they get a positive or negative test?

- - What is the expected pathway of care for self-testers that test positive? How often do we expect self-testers to seek care? What therapeutics are they likely to take for their symptoms at home? Are there data sources for this?
  - What is the expected pathway of care for self-testers that test negative? How often do we expect self-testers to seek care for their symptoms? Repeat testing? What therapeutics are they likely to take for their symptoms at home? Are there data sources for this?
- If someone has no access to self-testing at home, what self-medication are they likely to take? At what point are they likely to seek care? Is there any data on this?

1. *Treatment and care at health centers*

Thank you for sending your countries’ treatment and care guidelines. We want to better understand what care pathways are used in practice for confirmed positives, confirmed negatives, or suspect cases that cannot be tested:

- What range of monitoring, treatment and care options are commonly available for confirmed cases at different levels of severity/risk? What options are unlikely to ever become available in the future?
- Are any COVID therapeutics not recommended or recommended against by WHO (see [WHO recommendations on their website](https://www.who.int/publications/i/item/WHO-2019-nCoV-therapeutics-2022.5)) likely to be given to positive patients? How commonly? Is there any data source about that?
- What happens if a suspect case cannot be tested? What treatment or care would they be provided for different severity/risk levels?
- What is the testing/care pathway for people with a negative diagnosis?
- Are there circumstances in which you would still treat for COVID?
- What is the most common disease that could be confused with COVID in your context?

1. *Evolutions in 2023*: are important changes expected in 2023 in terms of e.g., the availability and cost of different tests, screening tools and therapeutics? Do we have sources for that information?
2. *Other issues:*

- If we get back to the draft care pathway, are there any other issues we have not yet discussed that needs to be addressed?
- In light of our discussion, which of the parameter estimates we provided in appendix appear realistic and are there any that you would like to see adjusted? Why?

# **Annex 3 – Information Sheet and Consent Form**

Experts’ Consultation on Rapid Diagnostic Test (RDT) – Supported COVID Care Pathways

Participant Information Sheet

## **Invitation and summary**

We would like to invite you to take part in our research study entitled: “Experts’ Consultation on Rapid Diagnostic Test (RDT)-Supported COVID Care Pathways”. Participation is entirely voluntary – there is no obligation to participate in the study and if you choose not to participate this will not affect your future career. Before you decide we would like you to understand why the research is being done and what it would involve for you. Please read the information carefully. You may ask us any question you may have about the research. Feel free to talk to others about the study if you wish.

This participant information sheet first describes the purpose of the study and what your participation will involve if you decide to take part. We then give you more detailed information about the conduct of the study. If you decide to take part in this project, you will be asked to sign the consent form that is available at the end of the information sheet.

This project is an online consultation on COVID screening, testing and care practices. We want to better understand how similar or different practices are in different regions of the world. The project will help us understand the benefits and costs of using rapid diagnostic tests (RDTs) to support COVID treatment and care as compared to standard of care without rapid testing. The goal is to help governments decide on their future priorities. If you participate in this study, you will be asked questions around practices in your country during two online discussion sessions of one and a half hours each with other experts from your region. Additionally, you will be offered to participate in a half hour joint meeting summarizing the results of the discussions with the different regions. Participants will be experts who understand COVID care practices in low- and middle-income countries from three regions; Africa, Asia and Latin America. All information provided by participants will be anonymized to ensure that specific contributions cannot be linked to an individual or a country.

## What does participation involve?

**Purpose and background of the research**

The purpose of this project is to better understand local guidelines and practices associated with screening, testing, monitoring, treating or caring for symptomatic patients that may have COVID. We want to understand what happens for patients that may have COVID and how their experience may differ if rapid diagnostic tests (RDTs) are available as opposed to if they are not. We will discuss both professional use RDTs delivered by health professionals and RDTs used for self-testing. This project will complement official guidance (e.g., from WHO) by giving us better insights on how field practices may differ from such guidance and what constraints are faced. It will also be complemented by data and information from literature reviews. The information will be used to inform an assessment of the cost-effectiveness of using RDTs to enhance COVID treatment and care. The scope of the project is not a specific country; instead we seek to understand the range of COVID-related practices and challenges in several low- and middle-income countries. We are consulting with a group of 8-12 experts with experience of COVID-19 care practices in their country. These experts represent a range of contexts from Africa, Asia and Latin America.

We would like to invite you to take part in our research as one of these experts.

**What participating would involve**:

If you take part in this research, you will be asked the following:

1. You will first be invited to send any relevant national document (guidelines, protocols, and specific data if available) that you may have to help guide future conversation.
2. You will then participate in two regional meetings lasting one and a half hours each. These meetings will group 3 to 4 experts per region (for the three regions of Africa, America and Asia). Experts participating in the consultation will be asked about:
   - In the first meeting: important population subgroups that we want to understand more about and should consider when analysing the costs and benefits of COVID-19 RDTs (this may relate, for example, to prevalent comorbidities in your country or particularly hard-to-reach patients that are unlikely to present to care centers), screening and testing practices, and any knowledge regarding uptake and linkage to care for self-testing patients.
   - In the second meeting: monitoring, treatment and care practices for patients with COVID-like symptoms in the presence/absence of rapid diagnostic tests (see updated [WHO recommendations on their website](https://www.who.int/publications/i/item/WHO-2019-nCoV-therapeutics-2022.5) for your convenience), foreseen evolution in 2023, feedback on a draft COVID care pathway and parameter estimates found through literature search (a draft pathway and summary of estimates will be sent in advance of the meeting).
3. A final half hour meeting will combine experts from all regions. This joint meeting will summarize inputs from the different regions and implications for our research and will provide you with an opportunity to hear what other regional groups have said.

There are no right or wrong answers to the questions you will be asked during the meetings. We want to hear many different viewpoints and would like to hear from everyone. We hope for honest answers even when your responses may not be in agreement with those of the rest of the group. Out of respect for each other, we ask that only one individual speak at a time in the group and that responses made by all participants be kept confidential. You may withdraw your participation from the meetings at any point.

We will capture a video- and audio-recording of the sessions, which we are taking for practical reasons as it can be difficult to accurately note what you say during the discussions, and will also keep a written record of the answers provided by participants. Both will be confidential, available only to study researchers. The video/audio recording will be kept for a maximum of a month, to give us time to ensure the accuracy of the written record. The written record will be kept for a maximum of two years. While participants in the meetings will know what other participants in the same group say, we will ask everybody to retain confidentiality regarding contributions from others. Any research output that we produce will keep your identity anonymous. For that purpose, we will not associate names of experts, institutions or countries with specific outputs and will ensure that no specific information enabling the identification of either you as an individual or the country you are speaking about is retained in any future publication.

Your inputs will contribute to the materials that may be subsequently used for publication(s) on the use of rapid diagnostic tests to support COVID-19 treatment and care. If you want, you can be included as part of a group co-authorship in the publication. In the same way as the decision to participate in this study is entirely voluntary, it is also entirely up to each participant to decide whether they want to be part of the co-authors of this research or not. Participants who choose to be co-authors will contribute to the writing of the manuscript and the final decision to submit for publication. If you decide to be a co-author of the research, we will still ensure that your specific contribution remains anonymous and that you cannot be linked to your specific contribution.

**Risks and benefits of participation:**

We do not anticipate that there are any risks associated with your participation, but feel free to contact us with any worry that you may have. Benefits of your participation may include a better understanding of practices in other countries and regions, and group co-authorship, if you so wish.

## **Additional information**

**Questions:** If you have any questions, do not hesitate to contact Gabrielle Bonnet: [gabrielle.bonnet@lshtm.ac.uk](mailto:gabrielle.bonnet@lshtm.ac.uk)

**What if something goes wrong?**

Concerns or complaints can be submitted to: Liz Corbett, [liz.corbett@lshtm.ac.uk](mailto:liz.corbett@lshtm.ac.uk)

**What will happen if I do not want to carry on with the study?**

Participants are free to withdraw from the process at any time without negative consequences or having to provide a reason. However, if you have already participated in one of the meetings, we may not be able to remove statements that you have already made as part of the broader discussion.

**What are my choices?**

You may choose to participate in the study or not. If you choose to participate, you may decide to become part of a group co-author within the publication directly resulting from this study. If you do so, your name and institution will appear in the study, but we will ensure that your contribution cannot be linked to you, your institution or your country specifically. Alternatively, you may wish to remain entirely anonymous, in which case we will ensure that your name and institution do not appear anywhere in the publication.

**Research team:**

The research team includes the following:

Professor Anna Vassall: [anna.vassall@lshtm.ac.uk](mailto:anna.vassall@lshtm.ac.uk)

Professor Mark Jit: [mark.jit@lshtm.ac.uk](mailto:mark.jit@lshtm.ac.uk)

Professor Liz Corbett: [liz.corbett@lshtm.ac.uk](mailto:liz.corbett@lshtm.ac.uk)

Assistant Professor Gabrielle Bonnet: [gabrielle.bonnet@lshtm.ac.uk](mailto:gabrielle.bonnet@lshtm.ac.uk)

**Funding:** this research is funded by UNITAID (a global health initiative that works with partners to bring about innovations to prevent, diagnose and treat major diseases in low- and middle-income countries) and population services international (PSI).

**Who has reviewed this study?**

All research involving human participants is looked at by an independent group of people, called a Research Ethics Committee, to protect your interests. This study has been reviewed and given favourable opinion by The London School of Hygiene and Tropical Medicine Ethics Committee.

Experts’ Consultation on Rapid Diagnostic Test (RDT) – Supported COVID Care Pathways

Consent to take part in consultation

Thank you for agreeing to take part in the above consultation. You will be invited to take part in experts’ consultations (see information form) and send us relevant written documentation in advance, if it exists.

Ethical procedures for academic research undertaken from UK institutions require that interviewees explicitly agree to being interviewed and to how the information contained in their interview will be used. This consent form is necessary for us to ensure that you understand the purpose of your involvement and that you agree to the conditions of your participation. Please therefore read the accompanying information sheet then, if you agree to participate, sign this form to certify that you approve the following:

- I……………………………………… voluntarily agree to participate in this research study.
- I understand that I am under no obligation to take part in this study and that, even if I agree to participate now, I can withdraw at any time or refuse to answer any question without any consequence of any kind.
- I have had the purpose and nature of the study and of my participation explained to me in writing in the Information Sheet and I have had the opportunity to ask questions about the study.
- I understand that I will not benefit directly from participating in this research.
- I agree to the consultation being video and audio-recorded and for the responses I give to be collated.
- I understand that all information I provide for this study will be treated confidentially and analyzed by the research investigators whose names are at the top of this form. Access to nominative information will be limited to these investigators and academic colleagues and researchers with whom they might collaborate as part of the research process.
- I understand that any summary content, or direct quotations from the consultation, that are made available through academic publication or other academic outlets will be anonymized so that I cannot be identified, and care will be taken to ensure that other information in the meeting that could identify me is not revealed.
- understand that signed consent forms and transcripts of meetings will be retained in electronic format for a period of two years from the end of the final meeting, while video and audio recordings used to ensure the accuracy of written records will be kept for a month after the end of the meeting at most. I will receive an electronic copy of this consent form and of the information sheet for my records.
- I understand that under freedom of information legalisation I am entitled to access the information I have provided at any time while it is in storage as specified above.
- Any modification to the conditions above will only occur with your further explicit approval.
- I understand that I am free to contact any of the people involved in the research to seek further clarification and information.

*Signature of research participant*

------------------------------------------- ----------------

Signature of participant Date

*Signature of researcher*

I believe the participant is giving informed consent to participate in this study

------------------------------------------- ----------------

Signature of researcher Date
